# Pathological Shoeing1Read at the June meeting of the Schuylkill Valley Veterinary Medical Association.

**Published:** 1897-09

**Authors:** W. H. Yingst

**Affiliations:** Shenandoah, Pa.


### PATHOLOGICAL SHOEING.[1]

By W. H. Yingst, V.S.,
SHENANDOAH, PA.

The subject of my paper, "Pathological Shoeing," is one that has attracted a great deal of attention, especially of late years, and the discussion has brought out a great variety of opinions. It is a subject of such a nature that, if one is interested, he will find that each day adds to his knowledge, and there is no saying how long a man may study and practice before becoming expert enough, by looking at a horse's foot, immediately to know the kind of shoe to apply to correct a certain fault. But I believe that by study and observation a man can acquire this faculty, and will recognize that the same faults in different horses cannot all be corrected by any one method.

The horny box contains the lower portion of the second and third phalanges, the navicular bones, and lateral elastic fibro-cartilages, covered by a vascular network allowing of but moderate movement, and synovials of the flexor-tendons, resting on an elastic cushion, all enveloped in a dense keratogenous box—wall, sole, and frog.

In the horny box, in front, besides the bones, are observed the anterior extensors of the phalanges; on the sides, the anterior and posterior lateral ligaments. The lateral cartilages occupy the space between the posterior border of the anterior lateral ligaments and the anterior borders of the posterior lateral ligaments. The perforans tendon, behind, reaches the navicular bone and spreads into a wide, keratogenous or horn-growing membrane. The plantar cushion is a fibro-fatty mass, in shape like a frog, and lies against the posterior portion of the phalanx, and corresponds to the faces of the lateral cartilages, and is continuous with their fibrous capsule; the inferior surface rests on the superior face of the frog; it is dense and elastic.

[1] Read at the June meeting of the Schuylkill Valley Veterinary Medical Association.

The horny box comprises the wall and sole. The wall, if spread out, would be a perfect crescent; the ends of the crescent turn in at an acute angle behind, being the heels, and extending obliquely forward and converging toward each other, comprise the bars; immediately above the heels can be observed the glomes. The outer wall is usually the thickest. At the upper extremity of the wall is a concave bevel (cutidural cavity), in front the periople or coronary band. In very young animals this band extends down the wall, but in the rasped hoof it has disappeared, except at the top of the wall, where it is thick and adherent.

Structure of horny box: On the inner side a series of laminæ, five to eight hundred in all (if spread out on a flat surface would probably cover a space of twelve square feet); the longest, corresponding to the length of the wall and one thirty-second to one-eighth inch in width. Next to the laminæ a layer of white cement, next a layer of dense fibrous tissue, then two more layers of fibres, increasing in density from within to without. Where the sole joins the wall the laminæ disappear, thus bringing the sole in direct apposition with the cement layer, which is always in excess where the sole joins the wall. The frog—a homogeneous, wedge-shaped structure—is continuous with the coronary band. In alluding to the white line I mean the cement layer, the bony column from the metatarsus or metacarpus to the third phalanx included.

To come at once to the subject of this paper, we will first consider a perfect foot and hoof—one upon which a horse travels perfectly sound. The bones are and must be in perfect apposition, and are a column. The front limbs are vertical with a line drawn from the point of the shoulder down. The os pedis is level and on the same horizontal plane with the sole, which itself is level. Quite a number of horses are brought for treatment that would travel square and sound if the hoof was level, which is essential, though most farriers seem to attach little importance to it, viz., if too much wall to the outside, the horse would stand wide, the os pedis would not be on the same horizontal plane as the ground, but the bones would represent a straight column, though they would be in an oblique direction from the body. In this case by simply making the foot level the animal would stand in a correct position and be made to travel square. The horse will always travel or stand in a position that will cause the bones to be a perfect column; thus he points, invariably, to the highest part.

The trouble with many farriers is that they do not understand the art of levelling and balancing, which is the first and most important knowledge. If the wall be pared, cut, or rasped to a level with the sole, the white line is plainly and evenly seen, and a level bearing is assured from the ground up—that is, of course, providing the structures in the hoof are normal. If the heels of the front hoof are too high, the horse will point back under himself. The hind feet may be perfect, but they are necessarily carried forward under the body to assist the balance. In this way the structure of the hind feet will become overtaxed, the horse lame in all four feet (an example where the good suffers with the bad).

In a case of sore-tendons all that may be required is to level the hoof. A centre-bearing shoe corrects nearly all faults, because, with such a shoe, the horse is allowed to rock and to seek the position most comfortable; he almost immediately ceases to point, and, though painfully lame before applying the centre-bearing shoe, an improvement will at once be noticed.

In a case where the hoof is found to be level and the horse points, one would at once know that the bottom of the hoof and the end of the column of bones are not on the same horizontal plane. Take, for example, a horse standing with the front feet ten inches or more apart, you would know at once that there is a greater space between the os pedis and sole to the outside than to the inside, and at the same time the bottom of the hoof is perfectly level. You will understand at once why the animal stands and travels so wide, viz., to cause the bones to stand as a straight column, though it is an oblique column. A horse travelling wide from the above cause usually interferes, striking the inside of the opposite leg a little above the fetlock-joint at each step. The farrier might wonder how a horse travelling so wide could interfere, but any intelligent veterinarian can readily comprehend: as the foot is lifted from the ground and the leg advanced the adductor muscles carry the foot in, giving it a semicircular movement, while at the same time the hoof rotates in and strikes the opposite foot, usually above the fetlock-joint. If this fault is due to an uneven hoof, levelling is all that will be required; but due to pathological changes as above described, or as often observed in the so-called awkward colt, caused by an unbalanced third phalanx, it is usually inherited. In such a case pare and rasp the hoof level, then apply a shoe elevated to the inside by means of two calks, placing one back of the inside toe and the other crossing midway the line of the inside. The horse will almost at once bring the limbs into a vertical position—*i. e.*, vertical at a line drawn from the point of the shoulder down—and the bones will be a perfect column. The horse will travel square, semicircular movement of the leg and foot and rotation of the hoof will cease, and, in consequence, will not strike.

It is common in some localities to see awkward colts—*i. e.*, cow-hocked—toes turned out of both front and hind limbs. The owner usually finds it necessary to use boots on his horse. In a case of this kind, where the animal straddles widely, the toes turned decidedly out, a modification of the above shoe will cause the animal to go square almost at once. It consists in, first, making the foot perfectly level, then applying a shoe provided with a calk to its inside, the calk to be two inches long. Instead of allowing the calk to be of a uniform thickness it is made an inclined plane, starting square and fully a half-inch thick in front, and gradually decreasing from before to behind, where it ends level with the shoe.

A horse perfectly balanced to-day may lose that nicety of equilibrium to-morrow, if, being reshod, a perfectly level bearing is not obtained. An enormous growth at the inside or outside, or at the toe or heel, will cause the horse to point, and always to the most elevated place—as he will also point toward the highest part of an uneven shoe, though the hoof be level. A perfectly sound horse can be made to stand in a number of strange positions at the will of the man who does the shoeing. By putting on a shoe elevated at the inside, if the calk is high enough, the animal will be made to cross his leg, which proves how easily a horse's balance can be affected. The various directions a horse will point, being too high at parts, are too numerous to mention. When a horse points it is to get relief for an unbalanced, tired phalanx, which would naturally cause pain if the horse would stand to the vertical line. Thus the trouble continues, the horse not always being able to take care of the structure of his foot, and, especially when travelling, becomes painfully lame, and goes from bad to worse—the much-reiterated "no foot, no horse," becomes a fact with most owners of such unfortunate animals. Why? Because the owner, after spending considerable money for shoeing, and as much more for medical treatment, is so disgusted that the horse is sold for a mere song to a carter. A good horse is thus sold and isolated from his proper sphere for the want of proper treatment.

To recognize an unbalanced third phalanx, and to be able to know the position of that bone in the horny box from the way the horse stands, is the problem to comprehend to be able to correct all faults, and in correcting any trouble a perfectly level hoof is essential; but if the third phalanx is unbalanced in a level hoof, you have an abnormality to deal with—one that has undergone pathological changes as above described. In such cases an instance of treatment has been mentioned.

An interesting way to make oneself understand the causes of different lamenesses and pointing from an unbalanced third phalanx, is to procure the third phalanx, have a stick eighteen inches long fastened to its pyramidal eminence, attach a string at the upper end of the stick, and put a weight on the lower end of the string. Place the third phalanx on a level and the stick will stand vertical; then by raising the third phalanx at given points, singly, by means of a ruler, one can readily perceive the different troubles that such a condition would cause in the living horse, and why the animal would point.

A thorough practical knowledge of the theory laid down in this paper will make a man so competent that he will not get a case of interfering which he cannot correct, and that, too, at the first dictation at shoeing.

Various kinds of shoes are used. The "ball-bearing" is an iron plate, the lower portion convex, representing part of a sphere. If this shoe is used on a horse painfully lame, from an unbalanced foot, a remarkable improvement will be noticed when the horse is led away from the shop.

Another shoe which allows the horse to seek the most comfortable position, whether standing or travelling, is the centre-bar shoe, made by having the bar run from the toe and resting behind on a bar crossing at the heels. The centre-bar is to be a decided rocker from before to behind. A centre-bearing shoe acts exactly like the ball-bearing. It is a cross at the centre of the shoe, and a calk placed exactly in the centre of the bar acts as a pivot.

To prevent a horse from forging, allow a long toe to the hind hoof, and there is an advantage in allowing the toe of the shoe to extend half an inch beyond the toe of the hoof. The front hoof should be perfectly level and as short at the toe as allowable. The shoe should have a decided roll from two inches back of the toe to the toe. I have frequently observed farriers, when treating forging, allow the wall of the hind hoof to extend beyond the toe of the shoe, giving as an excuse that if the horse would strike he would hit with the toe of the hoof and not with the shoe, and thus do less damage; but, of course, the latter method would not by any means remove the cause.

In contracted heels the hoof gets smaller, the frog becomes atrophied, there is an increased concavity of the sole, and the walls at the quarters and heels approach, and may become almost vertical. The outer surface of the wall is usually dry, and the hoof is predisposed to crack. A shoe fitting too tightly at the heel, and nails driven too far back, preventing the hoof from expanding normally when the weight of the body is placed upon it; allowing the shoe to remain on too long, the hoof getting an abnormal length and growing away from its vascular supply, causing it to dry out; rasping the walls, as practised by farriers, allowing of evaporation and diminishing elasticity; cutting the bars or opening the heels, as it is termed, are some of the easily recognized causes of contraction. Pare and rasp the hoof until the white line is plainly and evenly seen, and then the hoof will be at a proper length; turning the horse in the field, or wearing tips, if kept at work, are two remedies, but not always satisfactory to owners. The heels can be spread beautifully in four or five weeks, while the animal is at work, by means of the hoof-expander, the patent of the late Prof. David Roberge, of New York city. If the heels are to be spread by means of a bar-shoe, the bar should be the shape of a V, the point of the V resting well toward the centre of the frog.

For corns a shoe must be so constructed as to avoid the corn altogether, as toe- and quarter-cracks, when they occur, are usually a sequel to one of the above. I will not speak of a certain shoe to correct this trouble; predisposing causes must be dealt with, the hoof can be softened with oil, and then the crack completely cut out in the shape of a V; then if the hoof is kept soft, and its other troubles looked to, a sound hoof is assured.

Horses having heels that grow down vertically should receive special attention in keeping the heels at proper length, lest the horse becomes worthless. Such animals should be provided with a shoe that rocks backward. It is a cure as well as preventive.

Knee-spring can result from various troubles, but is usually caused by the hoof being too high to the inside, in which case the knees are noticed to be out as well as sprung forward; whereas, if caused by being too high at the heels or toes, the knees are noticed to be sprung straight forward. A horse becoming knee-sprung from an overgrowth at the inside of the hoof is subject to other troubles, such as bursa enlargements, spavin, and ring-bone, the too great height at the inside causing an excess of pressure upon the bones at the joints, producing inflammation and bony deposits. Nine horses out of ten suffering from inside spavin are too high at the inside, and usually toward the toe; therefore, by paring the hoof level and applying a shoe that has its inside toe cut down to a decided bevel-roll, the horse will frequently be made to go sound. The outside spavin, or any exostosis at the joints at the outside of the limb, is caused by the outside toe being too high, and naturally the treatment is the reverse of that for inside exostosis.

Knuckling of the hind feet is caused and cured in the same manner as knee-spring and bony deposits.

If a colt is watched and cared for, or I might say if all colts were looked to, in regard to keeping the hoofs pared to the proper length, all the troubles resulting from an unbalanced third phalanx would be avoided. This assertion can be verified by calling your attention to the colts and horses on Mr. Robert Bonner's farm. He always has a hundred or more, and takes particular care in keeping their hoofs at a proper length by paring and levelling whenever necessary. In consequence, he raises colts to horsehood, and not a faulty animal is found on his farm.

I might continue this paper to twice its length by dwelling on other lamenesses and methods of correcting the same; but I am fearful that I already have taken up too much time by explaining things that would be readily understood by the profession. I will reiterate that an unbalanced third phalanx is the cause of all trouble, and that knowing how to level a horse's hoof and to bring the phalanges into perfect apposition is the key to the only true method of pathological shoeing.

